# The Storage of Potable Water

**Published:** 1895-06-29

**Authors:** 


### The Storage of Potable Water.

One of the questions which has to be most carefully considered in devising a water supply for any large community is that of storage. The rainfall varies so greatly at different seasons that it is necessary to provide for periods of drought, either by artificial reservoirs, constructed usually at very great cost, or by taking care in selecting a gathering ground that the natural storage, due to the nature of the strata of which it is composed, shall be sufficient to render such appliances unnecessary; and there can be but little doubt that one strong point in favour of the use of the Thames and Lea as sources of water supply for the metropolis lies in the enormous capacity which their gathering grounds offer for the natural storage of water. This is well shown by Major-General Scott, in his Report to the Local Government Board, by a couple of diagrams, one of which indicates the monthly rainfall, and the other the average daily discharge of water at Teddington Weir for each month in the year. The minimum rainfall in 1893 was reached in April, and even in March it was very slight, yet the minimum of discharge at Teddington was not reached until September, the river having been for all the intervening months to a large extent fed by previously absorbed water. Even the great rainfall in July did not interfere with the gradually lessening discharge. Although the rainfall in July was greater than in any month except February and October, it did not produce any rise in the outflow, which continued to diminish until the October rains began to charge the soil and leave the surface less absorbent. Then, when the soil was full, the excess began to run away, and the outflow over the weir at Teddington rose steadily until February, when, coincidently with the greatest rainfall, there also occurred the maximum of discharge. It is very important to observe the great difference in the behaviour of a rainfall according as the land on which it falls is at the moment wet or dry, sodden or absorbent. After the dry summer, the rains of October and November, averaging over three inches each month, barely sufficed to produce an outflow of 500 million gallons per day, while a fall of 2·48 inches in December, when the ground had become charged, was straightway accompanied with an outflow of more than 1,000 million gallons per day, and a fall of 3·35 inches in February immediately ran off at the rate of more than 3,000 million gallons per day. If, then, we bear in mind that a nearly equal rainfall in July, viz., 2·85 inches, was accompanied with an outflow of only 396 million gallons, less in fact than during the preceding month, when the fall was quite trivial, we can see how entirely the summer rains are absorbed by the land, and how completely the constancy of the Thames supply depends on the storage of the water in the land rather than on the effect of the rain falling in any given month. This is the strong point made by those who advocate the retention of the Thames as a source of water supply for the metropolis. The portion of the basin of the Thames which is above the intakes of the water companies contains about 3,500 square miles and, over the larger portion of this area, is formed of permeable and water-bearing strata; and the dry-weather flow of the Thames is maintained by springs issuing from these strata, which act as enormous and never-failing water reservoirs.

This matter derives its importance from a consideration of the extremely small amount of artificial storage provided by the water companies after all the millions of money they have spent. It is stated in the Report before us that collectively they cannot impound more than seven days' supply at the present summer rate of consumption, and that, with the exception of the East London Company, they must take from the river or other sources day by day the quantity required by the population. Whatever else happens, then, in regard to the water supply of London, natural storage will have to be trusted to for maintaining the continuity of supply, and it will be hard indeed to find an area sufficiently large to collect the amount of water required, sufficiently pervious to store it, and at the same time so uncultivated and free from habitations that the water flowing from it in times of flood shall not be liable to contamination. If, then, such land cannot be found, we shall still have to depend for the purity of our water upon filtration, in which case, we are asked, why go further afield for the raw material when we already have it at our doors? The advice of the Royal Commission was, in effect, to stick to the Thames, but to provide sufficient storage to tide over times of flood.

